# Homeobox Is Pivotal for OsWUS Controlling Tiller Development and Female Fertility in Rice

**DOI:** 10.1534/g3.116.028837

**Published:** 2016-05-17

**Authors:** Fredrick Mwamburi Mjomba, Yan Zheng, Huaqing Liu, Weiqi Tang, Zonglie Hong, Feng Wang, Weiren Wu

**Affiliations:** * College of Life Sciences, Ministry of Education, Fujian Agriculture & Forestry University, Fuzhou 350002, China; † Key Laboratory of Genetics, Breeding, and Multiple Utilization of Crops (FAFU), Ministry of Education, Fujian Agriculture & Forestry University, Fuzhou 350002, China; ‡ Department of Plant, Soil, and Entomological Sciences, University of Idaho, Moscow, Idaho 83844; § Institute of Biotechnology, Fujian Academy of Agricultural Sciences, Fuzhou 350003, China

**Keywords:** rice, *OsWUS*, homeobox, female fertility, tillering

## Abstract

*OsWUS* has recently been shown to be a transcription factor gene critical for tiller development and fertility in rice. The OsWUS protein consists of three conserved structural domains, but their biological functions are still unclear. We discovered a new rice mutant resulting from tissue culture, which hardly produced tillers and exhibited complete female sterility. The male and female floral organs of the mutant were morphologically indistinguishable from those of the wild type. We named the mutant *srt1* for completely sterile and reduced tillering 1. Map-based cloning revealed that the mutant phenotypes were caused by a mutation in *OsWUS*. Compared with the two previously reported null allelic mutants of *OsWUS* (*tab1-1* and *moc3-1*), which could produce partial N-terminal peptides of OsWUS, the srt1 protein contained a deletion of only seven amino acids within the conserved homeobox domain of OsWUS. However, the mutant phenotypes (monoculm and female sterility) displayed in *srt1* were as typical and severe as those in *tab1-1* and *moc3-1*. This indicates that the homeobox domain of SRT1 is essential for the regulation of tillering and sterility in rice. In addition, *srt1* showed an opposite effect on panicle development to that of the two null allelic mutants, implying that the srt1 protein might still have partial or even new functions on panicle development. The results of this study suggest that the homeobox domain is pivotal for OsWUS function.

WOX (WUSCHEL related homeobox) proteins, a subgroup of homeodomain transcription factors specific for plants, are involved in several developmental processes of plants, such as embryonic patterning, stem cell maintenance, and organ formation (van der Graaff *et al.* 2009). *WUSCHEL* (*WUS*) is the founding gene of the *WOX* gene family, originally identified in *Arabidopsis* (*AtWUS*). It serves as a central regulator in the maintenance of stem cell identity in the central zone (CZ) of the shoot and floral meristems (Laux *et al.* 1996; Mayer *et al.* 1998). *AtWUS* interacts with other genes to regulate the maintenance of meristems at both the vegetative and reproductive stages of *Arabidopsis*, through a feedback loop mechanism involving at least the *CLAVATA 3* (*CLV3*) and *AGAMOUS* (*AG*) genes. It has been found that the development of shoot apical meristem (SAM) and floral meristem (FM) at the early stages is regulated by the WUS-CLV3 signaling pathway. In *Arabidopsis*, WUS is produced in the organization center (OC) of SAM and migrates to the stem cells of CZ (Yadav *et al.* 2011), where it binds to the promoter of *CLV3* to activate *CLV3* transcription (Brand *et al.* 2000; Schoof *et al.* 2000). The expression of CLV3 negatively regulates *AtWUS* expression in OC via the CLAVATA ligand-receptor system to maintain stem cells in a constant population (Brand *et al.* 2000). During floral development, the FM identity is controlled by the WUS-AG feedback loop. At the reproductive phase, *AtWUS* activates the expression of *AG* in the early stages of floral initiation. After stage 6 of floral development, the stem cells in FM are terminated by *AG* that represses *WUS* expression, which allows gynoecium differentiation (Lohmann *et al.* 2001; Lenhard *et al.* 2001). A mutation in *wus-1* causes shoot meristem failure after seed germination and the mutant plants repetitively initiate defective shoot meristems, leading to many disorganized bunches of leaves at the base and the tip of stems. The flower of *wus-1* lacks most of the central organs and prematurely terminates in a single central stamen. *AtWUS* has also been found to regulate the anther and ovule development (Deyhle *et al.* 2007; Groß-Hardt *et al.* 2002; Sieber *et al.* 2004).

*OsWUS* in rice has not been subjected to intense studies until recently. An early report using *in situ* RNA hybridization has suggested that *OsWUS* is expressed in young leaf primordia with a prominent expression pattern in the lateral leaf margins (Nardmann and Werr 2006). Different from the expression pattern of *AtWUS* in *Arabidopsis*, the presence of *OsWUS* transcripts in the SAM of rice appears to be transient. During the later stage of development, *OsWUS* is expressed at the abaxial face of the emerging axillary meristems (AMs). Two recent reports on rice mutants defective in tillering have demonstrated that *OsWUS* is required for AM initiation and is not associated with the maintenance of the SAM (Tanaka *et al.* 2015; Lu *et al.* 2015). Both *OsWUS* mutants, *tab1-1* (Tanaka *et al.* 2015) and *moc3-1* (Lu *et al.* 2015), exhibit severe defects in tillering and flower development. Mutant plants produce no tiller and form a small panicle in the main culm. The floral structures are defective in some flowers of *tab1-1*, but look normal in all the flowers of *moc3-1*. The fertility defect in *moc3-1* is believed to be female sterile, but the cause of sterility in *tab1-1* has not been investigated.

The homeobox domain of WUS proteins has been implicated in DNA binding, an essential function of a transcriptional factor. It is a highly conserved motif with about 60−66 amino acid residues folded into a helix-loop-helix-turn-helix structure, which serves as a classic and specific DNA binding domain (Pabo and Sauer 1984; Gehring *et al.* 1994). Target genes of the WUS transcriptional factor in *Arabidopsis* have not been identified. The C-terminal structures of AtWUS, including an acidic domain, a WUS box, and an EAR-like motif, have been shown to be necessary for its biological function (Kieffer *et al.* 2006). The acidic domain serves as a transcriptional activator, while the WUS box is a repressor essential for all the activities of AtWUS (Ikeda *et al.* 2009). The EAR-like motif is conserved in plants and appears to be involved in transcriptional repression (Ohta *et al.* 2009). These multifunctional domains allow AtWUS to act as a bifunctional transcriptional factor, serving mainly as a repressor in stem cell regulation and becoming an activator for *AG* gene expression in floral patterning (Ikeda *et al.* 2009).

Three (homeobox, WUS box, and EAR-like motif) of the four domains of AtWUS are conserved in OsWUS (Nardmann and Werr 2006). The exact functions of these domains in OsWUS have not been investigated. In this study, we discovered a new independent mutant that forms large panicles with deformed and sterile spikelets and produces a drastically reduced number of tillers per plant. We refer to the mutant as *completely sterile and reduced tillering 1* (*srt1*). We applied a pooled whole-genome sequencing approach to map and clone the candidate gene and found that a 21-bp deletion of *OsWUS* was responsible for the mutant phenotype. Compared with the two published null mutants *tab1-1* and *moc3-1*, srt1 retains a complete WUS box and EAR-like motif as in the wide-type WUS protein. Phenotypically, *srt1* produced few tillers and was completely female sterile, suggesting that the homeobox is essential for tillering development and female fertility in rice.

## Materials and Methods

### Plant materials and field experiments

The main plant materials used in this study included two rice (*Oryza sativa* L. ssp. indica) cultivars Minghui86 (MH86) and 93-11, and the *srt1* mutant and its corresponding heterozygote (abbreviated as *srt1*-het) isolated from a population of plants regenerated from tissue culture of MH86. The mutant allele was inherited through *srt1*-het because homozygous plants of the mutant were sterile. Two F_2_ populations with segregation at the *SRT1* locus were developed from crosses of *srt1*-het (♀) × 93-11 (♂) and MH86 (♀) × *srt1* (♂). F_1_ individual plants were allowed for selfing to produce F_2_ seeds. Rice plants were grown in paddy fields under normal growth conditions in Fuzhou city, China. Several traits including the number of tillers per plant, length of panicle, number of primary branches, and number of secondary branches were investigated. Student *t*-test was performed in statistical analysis.

### Fast mapping of SRT1 using bulked segregant analysis by sequencing (BSA-seq)

From the F_2_ population of *srt1*-het × 93-11, leaf samples of 50 mutant plants (M-pool) and 50 phenotypically normal plants (N-pool) were collected and bulked respectively for genomic DNA extraction using the CTAB method (Murray and Thompson1980). Paired-end DNA-seq libraries with an average insert size of 400 bp were constructed for each pool using the Illumina TruSeq DNA LT kit (ID: FC-121-2001) according to the manufacturer’s instructions (Illumina, San Diego, CA). The libraries were sequenced on Illumina HiSequation 2500, each generating 4.6 Gb DNA sequencing data, representing an average of ∼12× coverage of the rice genome. In addition, Illumina sequencing data (unpublished data) of the genomes of MH86 and 93-11 with a coverage of ∼50× and 30×, respectively, were available from our previous work conducted at the Institute of Biotechnology, Fujian Academy of Agricultural Sciences.

Adaptor-trimmed reads from the four sequencing datasets (M-pool, N-pool, MH86, and 93-11) were aligned to the reference genome of rice (*Oryza sativa* L. ssp. japonica) cultivar Nipponbare (version IRGSP-1.0) using BWA (Li *et al.* 2009a) with default parameters. The uniquely mapped and properly paired alignments from the four datasets were picked by our custom Perl scripts and sorted by SAM tools (Li *et al.* 2009b). The alignments were then imported into FreeBayes (Garrison and Marth 2012) for identification of variants, including single nucleotide polymorphisms (SNPs) and short insertion/deletion (InDels), using default parameters. A subset of these variants, exhibiting explicit polymorphisms between MH86 and 93-11 and having a mean value of allele frequencies in the two pools ranging from 0.3 to 0.7, were selected as markers for genome typing. Based on these markers, a cubic average allele frequency difference (CAAFD) profile was plotted by scanning the whole genome with a 2000-kb sliding window at a step length of 10 kb. The CAAFD of a window was represented by its center point and was calculated with the following formula:

$$\text{CAAFD}={\left\{\frac{1}{n}\sum_{i=1}^{n}(\frac{m_{1i}}{m_{1i}+m_{2i}}-\frac{n_{1i}}{n_{1i}+n_{2i}})\right\}}^{3}$$

where *n* is the total number of markers in the window; *m*_1_*_i_* and *m*_2_*_i_* are the counts of the alleles from the genomes of MH86 and 93-11, respectively, at the *i*^th^ marker in the M-pool; *n*_1_*_i_* and *n*_2_*_i_* are the counts of the alleles from the genomes of MH86 and 93-11, respectively, at the *i*^th^ marker in the N-pool. The highest peak of CAAFD was considered to be the target region harboring *SRT1*.

### Fine mapping of SRT1

A set of InDel and SNP markers were developed in the target region according to the genomic sequence data of the two parents (MH86 and 93-11). These markers were then used to genotype 199 mutant plants selected from the F_2_ population of *srt1*-het × 93-11. Primers of the markers were designed using Primer 3 (http://bioinfo.ut.ee/primer3-0.4.0/) and were synthesized by Shanghai Sangon Biological Engineering & Technology Company (Shanghai, China). PCR for the markers was performed in a 15-μl reaction mixture containing 20−50 ng genomic DNA, 1× PCR buffer (10 mM Tris-HCl, pH 8.4, 50 mM KCl, 1.5 mM MgCl_2_), 200 μM of each dNTP, 0.5 μM each of forward and reverse primers, and 1 U of *Taq* polymerase under the following cycling conditions: 5 min at 94°, 30 cycles of 30 sec at 94°, 30 sec at 55°, and 1 min at 72°, followed by final extension for 5 min at 72°. PCR products were separated using 6% nondenaturing PAGE (300 V, 2 hr) and visualized by silver stain following the method of Xu *et al.* (2002) with minor modifications. Analysis of genetic linkages between the molecular markers and the *SRT1* locus was performed using MapMaker EXP 3.0 (Lander *et al.* 1987) with an LOD threshold of 3.0. The Kosambi mapping function was chosen to convert recombination rate into map distance.

### Pollen viability assay

To evaluate pollen viability of the wild-type and mutant plants, developing panicles were collected and stained with I_2_-KI staining buffer containing 1% I_2_ and 3% KI on a glass slide for 20−30 min at room temperature. The stained pollen grains were observed and photographed using a light microscope.

### Multiple sequence alignment

Amino acid sequences of AtWUS, OsWUS, and 12 other WUS-related proteins (WOX) in rice were downloaded from UniProt (http://www.uniprot.org/uniref/). Names of rice WOX proteins were adopted as proposed by Zhang *et al.* (2010). Multiple sequence alignment was performed using Clustal W (http://www.ebi.ac.uk/clustalw).

### Protein 3D modeling

Three-dimensional images of the mutant and the wild-type OsWUS proteins were obtained by simulation of the amino acid sequences using bioinformatics software (Chen *et al.* 2006).

### Data availability

The authors state that all data necessary for confirming the conclusions presented in the article are represented fully within the article.

## Results

### Greatly reduced number of tillers in srt1

**Figure 1:**
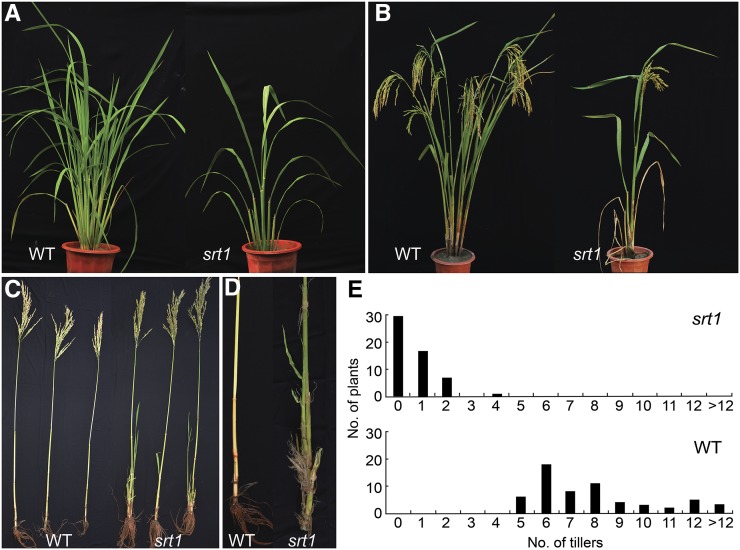
Growth and reproduction phenotypes of *srt1*. (A) Phenotype of *srt1* at the tillering stage. The parental cultivar MH86 served as the wild-type (WT) control. (B) Phenotypes of *srt1* at the ripening stage. The *srt1* mutant plant produced only one panicle that was completely sterile. (C) Defective tillers of *srt1*. Three tillers separated from a wild-type (WT) plant all produced a panicle. Three main culms from three individual *srt1* mutant plants all produced no valid tiller. Panicles were formed only in the main culms of *srt1*. (D) Highlights of the basal portions of stem of a WT tiller and a *srt1* main culm. Note adventitious root generated from the basal stem nodes of *srt1*. (E) Frequency of plants with various numbers of tillers. Note that most of the *srt1* plants had no tiller and some had one to four tillers per plant. All the wild-type plants produced more than five tillers per plant.

The mutant displayed a monoculm phenotype in most cases. Only a small percentage of the mutant plants generated a few (1−4) tillers (Figure 1). Before the fourth leaf stage, seedlings of the mutant and MH86 (wild-type control) appeared normal and indistinguishable. At the tillering stage, MH86 produced an average of 9.0 ±3.6 tillers per plant, whereas the mutant had only 0.5 ± 0.7 tillers per plant (Figure 1, A and E; see also Figure 3B). The base of MH86 plants was round-shaped because the tillers emerged from all directions. In contrast, the base of the mutant plants was flat, because the leaves emerged only from two opposite directions of the plant, forming a flat structure consisting of the main culm with sheaths and rare tillers if any. At the heading stage, MH86 plants had a panicle from every valid tiller, while most of the mutant plants produced only one panicle from the main culm (Figure 1B). The rare tillers that were formed from the basal nodes of the main culm of the mutant plants remained small and weak, and most of them could not produce panicles at the ripening stage (Figure 1, C and D).

### Complete female sterility in srt1

**Figure 2:**
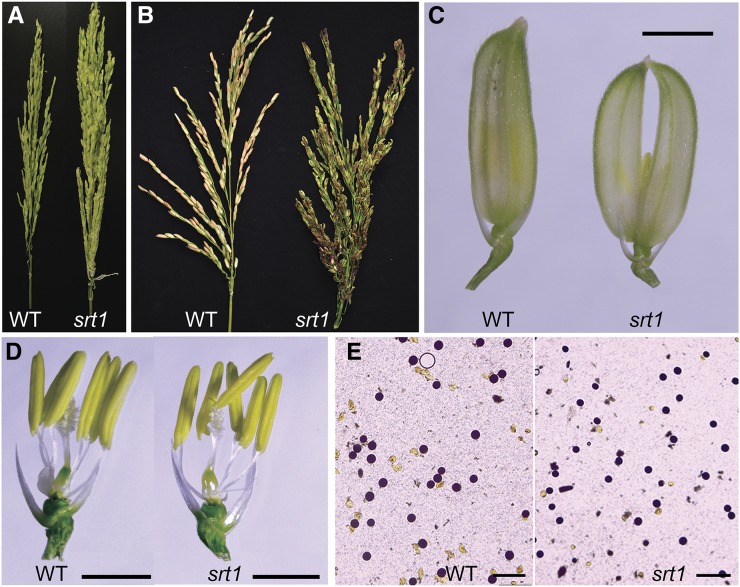
Reproductive phenotypes of *srt1*. (A−B) Comparison of young (A) and mature panicles (B) between MH86 (WT) and *srt1* mutant. Note that panicles of *srt1* were larger than those of WT, but produced no filled seed at all. (C) Some spikelets of *srt1* had deformed lemma and palea and could not close tightly. Bar = 5 mm. (D) Floral organs of spikelets with the lemma and palea detached were indistinguishable between *srt1* and WT. Bar = 2 mm. (E) Pollen grains of *srt1* were as viable as those of WT, when stained with iodine-potassium iodide solution. Bar = 200 μm.

**Figure 3:**
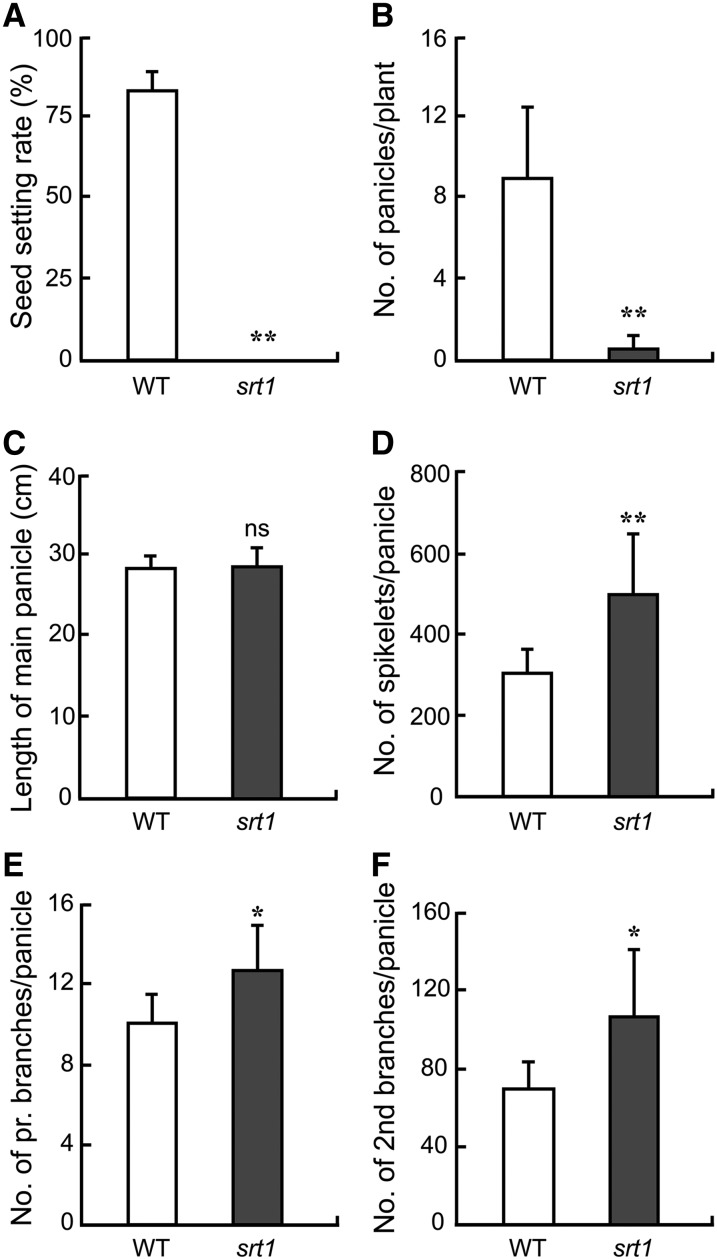
Panicle phenotypes of *srt1* and its parental cultivar MH86 (WT). (A) Seed setting rate (%). (B) Number of panicles per plant. (C) Length of the main panicle (cm). (D) Number of spikelets per panicle. (E) Number of primary branches per panicle. (F) Number of secondary branches per panicle. Asterisks indicate significant differences at *P* < 0.05 (*) or *P* < 0.01 (**) according to *t*-test. Error bar represents SD. *n* = 10. ns, not significant.

The heading time of the mutant was the same as that of MH86, but the panicle morphology of the mutant was different (Figure 2). There was no significant difference in panicle length between the mutant and MH86 (Figure 3C), but the panicle architecture of the mutant was different from that of MH86 (Figure 2, A and B), due to the increased numbers of spikelets, primary branches, and secondary branches in the mutant (Figure 3, B−F). In addition, the shapes of lemma and palea of the mutant spikelet were deformed and could not close tightly (Figure 2C). The spikelets of the mutant remained open at maturity, some anthers failed to dehisce at the flowering stage, and some anthers became completely twisted before emerging from spikelets. However, the inner floral organ structure of the mutant spikelets was similar to that of the control, containing six anthers and a pistil with a swollen base and two open stigmas (Figure 2D).

The mutant exhibited complete sterility (Figure 3A). To answer if the mutant was male sterile, female sterile, or both, we examined the male and female gamete fertility at the flowering stage. Pollen grains were stained with iodium-potassium iodide (I_2_-KI) solution. The size, morphology, and staining pattern of pollen grains were indistinguishable between the mutant and MH86 (Figure 2E), suggesting that the male gametes of the mutant are likely normal. To test the viability of male and female gametes of the mutant, we performed reciprocal crosses between the mutant and MH86. When the mutant pollen (♂) was used to pollinate eight independent male-sterilized panicles of MH86, seeds could be obtained from every panicle at an average seed setting rate of 9.9% ± 6.8%. However, when six male-sterilized panicles of the mutant were used as the maternal recipient (♀) to receive pollen grains from MH86, no single seed was obtained from 466 cross-pollinated spikelets (Table 1). This cross-pollination test clearly demonstrates that the male gametes (pollen grains) of the mutant are viable and the female gametes are completely defective.

**Table 1:** Seed setting rates of reciprocal crosses between *srt1* and its parental cultivar MH86

Cross(♀/♂)	No. of Panicles	No. of Spikelets^a^	No. of Hybrid Seeds^b^	Seed Setting Rate (%)^c^
MH86/*srt1*	8	859	77	9.9 ± 6.8
*srt1*/MH86	6	466	0	0

a Total number of spikelets from the panicles in the cross.

b Total number of F_1_ hybrid seeds from the panicles in the cross.

c Mean ± SD of seed setting rates of panicles in the cross.

### A recessive sporophytic mutation

F_1_ plants of MH86 × *srt1* showed normal growth and reproduction phenotypes indistinguishable from those of the control MH86, suggesting that the *SRT1* mutation was a recessive allele. When the F_1_ plants were allowed to self-pollinate, the resulting F_2_ progeny was segregated into 262 fertile and 71 sterile plants, which fitted a 3:1 ratio (χ^2^ = 2.21, *P* = 0.137), further confirming that the *SRT1* mutation was a typical recessive Mendelian trait. The fact that the F_2_ population exhibited a clear segregation ratio of 3:1 for fertile plants (+/+ and −/+) and sterile plants (−/−) from self-pollinated F_1_ (−/+) implied that *srt1* was a recessive sporophytic mutation.

### Cosegregation of sterility and tillering defects in srt1

In the F_2_ population described above, all the fertile plants exhibited normal tillering phenotype as the control MH86, and all the sterile plants had tillering defects, none having more than four tillers per plant. Among the 71 sterile mutants, 47 plants remained as monoculm with no tiller at all and the remaining 24 plants had only 1−4 tillers per plant, which were significantly fewer than those of the normal fertile plants under the same growth condition. This observation suggests that both defective traits, sterility and reduced tillering, were linked completely and controlled by the same *srt1* mutation.

### Mapping of SRT1

**Figure 4:**
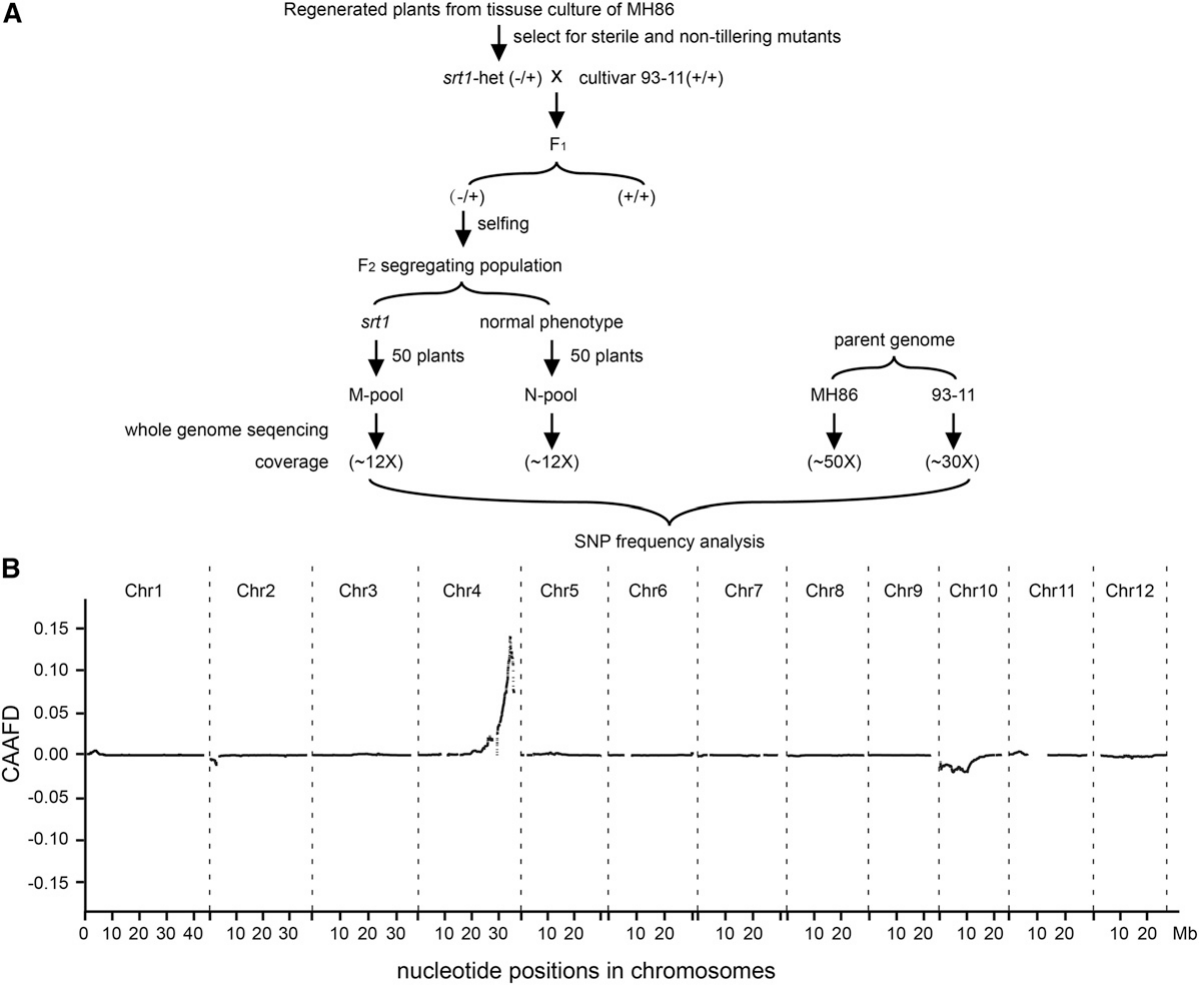
Mapping of *srt1* by BSA-seq. (A) Outlines of the procedures for BSA-seq. *srt1*-het(−/+) plants were crossed with cultivar 93-11 (*O. sativa* L. indica). The F_2_ mapping population was obtained from the F_1_ plants heterozygous at the *srt1* locus (−/+). Two genomic DNA pools were generated: the M-pool contained bulked DNA from 50 *srt1* mutant plants and the N-pool was bulked from 50 F_2_ plants that showed normal phenotypes. The two DNA pools were sequenced with ∼12× genomic coverage using Illumina sequencing strategy. Parental cultivars MH86 and 93-11 were sequenced with a coverage of ∼50× and ∼30×, respectively. The presence of SNPs and short InDel markers in the DNA pools was identified by genomic sequence comparison between MH86 and 93-11. (B) CAAFD (cubic average allele frequency difference) profile across the rice genome. One CAAFD peak was found in a region of ∼5 Mb (∼29–34 Mb) on chromosome 4.

A total of 656,084 variants (SNPs and short InDels) were selected as markers for mapping *SRT1*. Analysis showed that there was a major peak of CAAFD at the end of the long arm of chromosome 4, covering a region of ∼5 Mb (from ∼29 Mb to 34 Mb in the physical map), with the climax at ∼32.8 Mb (Figure 4). This region is evidently very likely to contain the causal gene of the observed mutation.

**Figure 5:**
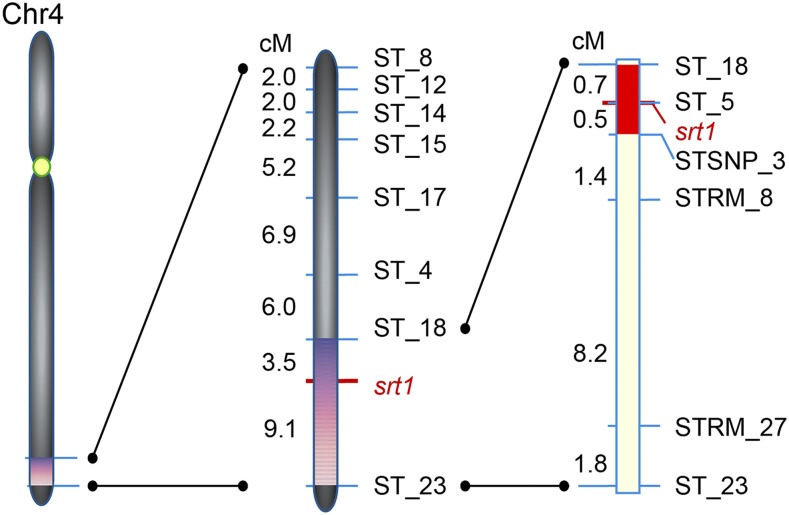
Fine mapping of *SRT1*. To fine map the *SRT1* locus, we compared the sequencing data and developed a set of InDel markers in the 5 Mb region of chromosome 4 identified by BSA-seq. Linkage analysis using these molecular markers first placed *SRT1* on a short genomic region between ST_8 and ST_23. Further linkage analysis using markers developed in this region delimited *SRT1* within a region of ∼231 kb between ST_18 and STSNP_3, with tight linkage to ST_5. Genetic distances in cM between two neighboring markers are indicated on left side of chromosome.

To fine map the target gene *SRT1*, we developed a set of InDel markers in the target 5 Mb region using the information from the sequencing data. By genotyping the 199 mutant plants from the F_2_ of *srt1*-het × 93-11 with these InDel markers, we narrowed down the *SRT1* interval from 32.9 cM (between ST_8 and ST_23) to 12.6 cM (between ST_18 and ST_23) (Figure 5). Further linkage analysis using markers developed in this region delimited *SRT1* within an interval of 231 kb between ST_18 and STSNP_3, tightly linked to ST_5 (Figure 5).

### Identification of srt1 as LOC_Os04g56780

**Figure 6:**
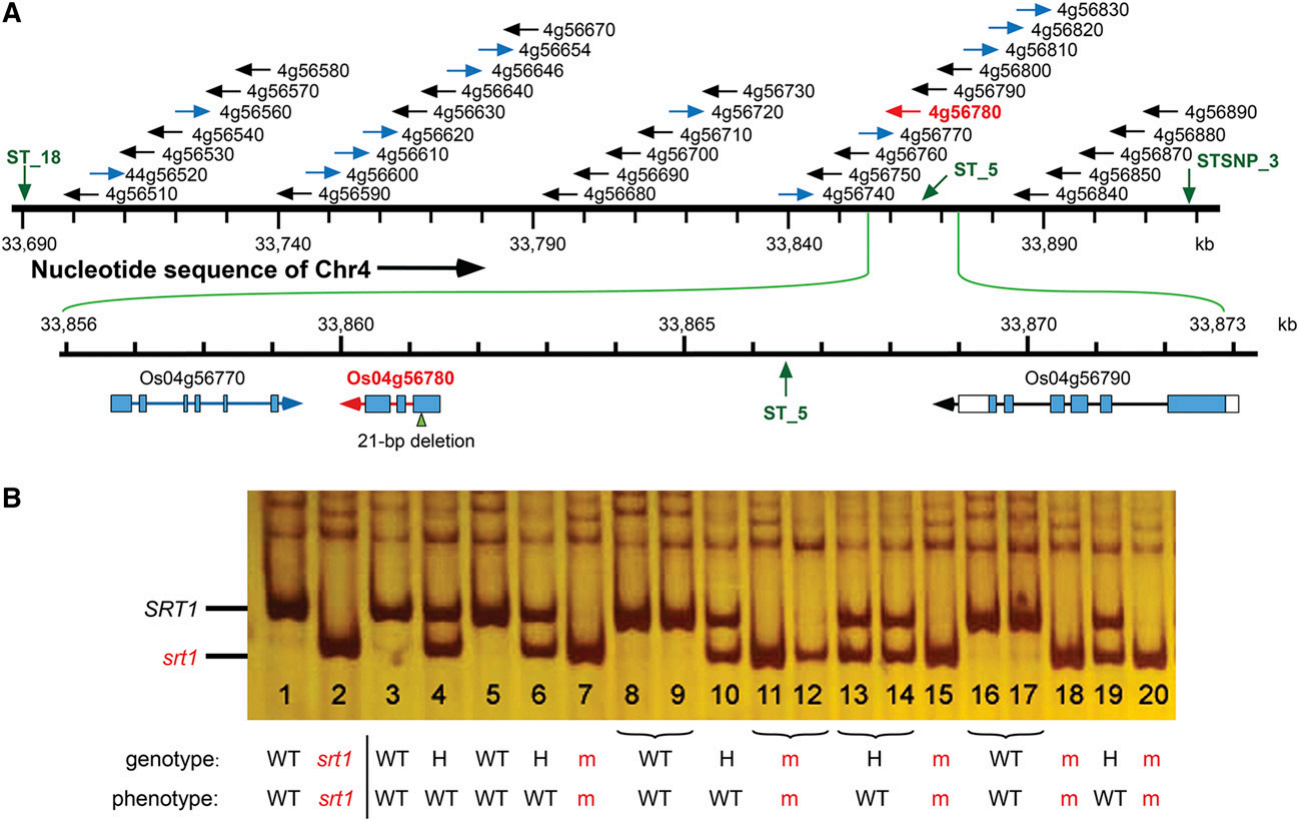
Identification of *SRT1* as a deletion mutant of *OsWUS*. (A) The 231-kb genomic region between ST_18 and STSNP_3 contains 37 coding sequences (CDSs). The directions and gene ID of these 37 CDSs are depicted above the chromosome 4 nucleotide sequence. Analysis of the genome sequencing data in this region detected only a 21-bp deletion in *LOC_Os04g56780* in the M-pool. InDel marker ST_5 was found ∼5 kb upstream of the start codon (ATG) of *LOC_Os04g56780*. No nucleotide change was found in all other 36 CDSs. Black/blue arrows indicate the directions of CDSs opposite to/the same as that of the chromosome 4 nucleotide sequence. The red arrow highlights *LOC_Os04g56780* as the candidate gene for *SRT1*. The exon−intron structures of *LOC_Os04g56780* and its two neighboring CDSs are depicted below the chromosome 4 nucleotide sequence. (B) A pair of primers (Loc-780-4) flanking the 21-bp deletion of *LOC_Os04g56780* was used for genotyping 280 F_2_ plants of MH86×*srt1* by PCR amplification. Lanes 1 and 2: positive controls of genomic DNA from *srt1* mutant and WT cultivar 93-11; lanes 3, 5, 8, 9, 16, and 17: genomic DNA samples from the mutant plants of the F_2_ population; lanes 4, 6, 7, 10−15, 18-20: genomic DNA samples from the phenotypically normal plants of the F_2_ population. Note that the 21-bp deletion at *LOC_Os04g56780* cosegregated with the mutant phenotype.

Analysis of the reference rice genome revealed that the 231-kb genomic region between ST_18 and STSNP_3 contained 37 open reading frames (ORFs) (Figure 6). To identify the candidate gene, we compared the assembled genomic sequences of this region among the M-pool, N-pool, MH86, and 93-11. We reasoned that the allelic variation (wild type “+” *vs.* mutant “−”) at the *SRT1* locus would appear as “−” only in the M-pool, both “+” and “−” in the N-pool, and only “+” in MH86 and 93-11. Of the 37 ORFs in this genomic region, only one allelic variation was found to meet the criterion, namely, a 21-bp deletion in gene *LOC_Os04g56780* (Figure 6A). In addition, the InDel marker ST_5 that was tightly linked to *SRT1* was found to be present ∼5 kb upstream of the start codon (ATG) of *LOC_Os04g56780* (Figure 6A). Furthermore, for a random sample of 280 plants from the F_2_ population of MH86 × *srt1*, a pair of primers (Loc-780-4F: CAACGTACCAGCTGCTGTAG and Loc-780-4-R: ATCAGGTCGCCCAACTCG) flanking the 21-bp deletion of *LOC_Os04g56780* was able to detect the deletion as homozygous mutant (−/−) in all sterile F_2_ plants, and heterozygous state (−/+) or homozygous wild-type state (+/+) in all fertile F_2_ plants (Figure 6B), suggesting cosegregation of the 21-bp deletion in *LOC_Os04g56780* with the mutant phenotype. Taken together, these three lines of evidence allow us to conclude that *LOC_Os04g56780* is the candidate gene of *SRT1* and the 21-bp deletion is the mutation that causes the mutant phenotypes.

### Mutation in homeobox of OsWUS

**Figure 7:**
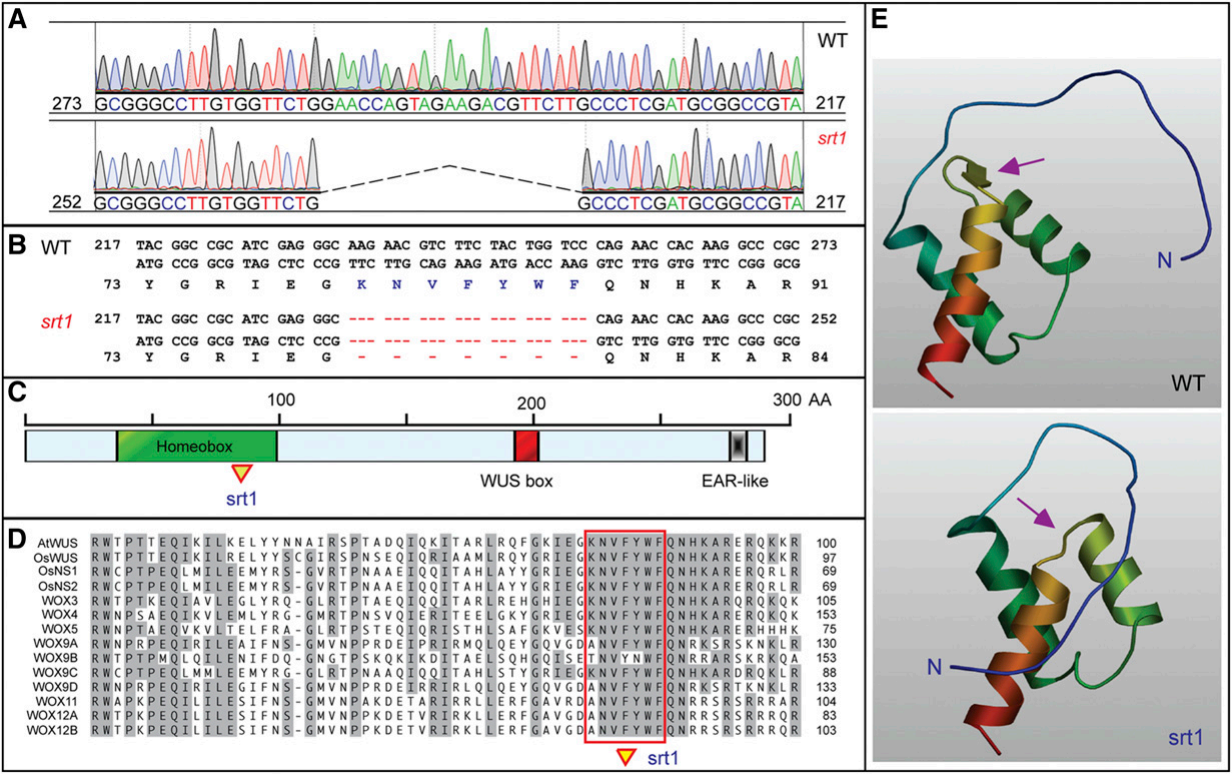
Identification of *srt1* as a deletion mutant of *OsWUS*. (A−B) The 21-bp deletion occurs in exon 1 of *LOC_Os04g56780*, resulting in deletion of seven amino acid residues in the highly conserved homeobox. (C) *LOC_Os04g56780* encodes the rice ortholog (OsWUS) of *Arabidopsis* Wuschel (AtWUS). OsWUS contains a homeobox (HD), a WUS box, and an EAR-like motif. Homeobox is highly conserved in the WUS and WUS-like (WOX) protein family. The WUS box motif is defined as a T-L-[DEQP]-L-F-P-[GITVL]-[GSKNTCV]. The ERF-associated amphiphilic repression (EAR) motif is defined as L-[ED]-L-[RST]-L. (D) Alignment of the HD domains of the 13 WOX family members in rice and *Arabidopsis* WUS (AtWUS). Boxed is the seven amino acids that were deleted in *srt1*. (E) 3D structures of the OsWUS N-terminal 100 residues including the variable N-terminus and the highly conserved HD domain. The seven-amino acids deletion in the mutant OsWUS protein results in lack of a short β-strand (purple arrow) in OsWUS.

*LOC_Os04g56780* encodes the rice ortholog (OsWUS) of *Arabidopsis* WUSCHEL (AtWUS), a key transcription factor that regulates stem cell maintenance in SAM and floral formation in FM (Laux*et al*. 1996; Mayer *et al.* 1998). *OsWUS* contains three exons and encodes a protein of 290 amino acids. Like other members of the WUS transcription factor family, OsWUS contains a homeobox domain (HD), a WUS box, and an ethylene-responsive element binding factor-associated amphiphilic repression (EAR)-like domain (Ohta *et al.* 2009). The 21-bp deletion occurs at exon 1 of *OsWUS* and would result in deletion of seven amino acids in the highly conserved HD (Figure 7, A−D). Analysis of three-dimensional (3D) models of the N-terminal 100 amino acids of OsWUS and the *srt1* mutant proteins revealed that the seven-amino acid deletion in the mutant results in lack of a short β-strand in OsWUS (Figure 7E).

## Discussion

In this study, we have identified a new mutant of *OsWUS* named *srt1*, which would produce a nearly full-length WUS peptide except for the deletion of seven amino acids in the HD. The mutation resulted in a dramatic reduction of tillers and complete female sterility. In addition, the mutation also caused morphological alterations in panicles and spikelets. Our findings suggest that *OsWUS* is an important gene with pleiotropic effects on plant development in rice.

As a new mutant allele of *OsWUS*, *srt1* is quite different from *moc3-1* and *tab1-1*, two other mutants of *OsWUS* reported recently (Lu *et al.* 2015; Tanaka *et al.* 2015). Both moc3-1 and *tab1-1* are truncated at the whole C-terminal half of the peptides, and are expected to completely lose their biochemical functions as transcription factors. As compared to them, srt1 has only a short deletion (∼2.4% of the whole peptide) and probably retains partial functions. Hence, although two other mutants of *OsWUS* have already been reported, the results of this study are still highly valuable and useful for our understanding of the biochemical functions of different domains of WUS proteins. The data presented in this report not only confirm and clarify the important functions of *OsWUS* in the regulation of tillering and flower development, but also unveil potential function of the HD of OsWUS in rice.

### The functions of OsWUS

Our observations have clearly shown that the *OsWUS* mutation affects four major traits of rice: tillering, fertility, panicle development, and spikelet development. As compared with two other recent reports, the main common defect found in all three mutants of *OsWUS* (Table 2) is tillering inhibition. Hence, controlling tiller development is undoubtedly the most significant function of *OsWUS* in rice. The incomplete inhibition of tillering in *srt1* implies that the srt1 protein might still have a partial activity.

**Table 2:** Phenotype comparison among *srt1*, *tab1-1*, and *moc3-1*

Mutant	*srt1*	*tab1**-**1*	*moc3-1*
Tiller number	0.5 (0∼4)	0	0
Panicle architecture	Denser	Shorter branch	Smaller
Flower structure	Defect in lemma and palea	Defective organs in partial flowers	Normal
Seed setting rate	0	Unknown	0
Fertility	Female sterility	Unknown	Female sterility

Although the sterility phenotype of *tab1-1* has not been fully observed and described, female sterility appears to be a common defect in *OsWUS* mutant lines (Table 2). Lu *et al.* (2015) speculate that *moc3-1* is likely to be female sterile. In our study, using reciprocal cross tests and pollen staining, we confirmed that *srt1* is female sterile. Hence, we conclude that *OsWUS* controls the female fertility in rice.

Like *srt1*, both *moc3-1* and *tab1-1* exhibit morphological changes in panicles as compared with the wild type (Table 2). However, while *moc3-1* and *tab1-1* panicles are smaller with fewer branches and spikelets than the wild-type control, panicles of *srt1* were larger and produced more branches and spikelets than the wild-type control. The opposite effects on panicle development between *srt1* and the other two mutants further imply that the srt1 mutant protein might still maintain a partial activity, or even have acquired a new function.

The effect of *OsWUS* mutation on spikelet development does not appear to be consistent among the three mutants (Table 2). In our study, we found that *srt1* spikelets developed deformed lemma and palea and could not close tightly, but their inner floral organs seemed indistinguishable from those of wild type (Figure 2C). By contrast, only some *tab1-1* spikelets show abnormal phenotypes (lack of one or more floral organs), and all *moc3-1* spikelets appear morphologically normal. The discrepancy among these results has prompted a question whether or not *OsWUS* is really involved in the regulation of spikelet development in rice. Perhaps, the function of *OsWUS* on spikelet development is dependent on the genetic background of the mutants.

### The function of homeobox in OsWUS

**Figure 8:**
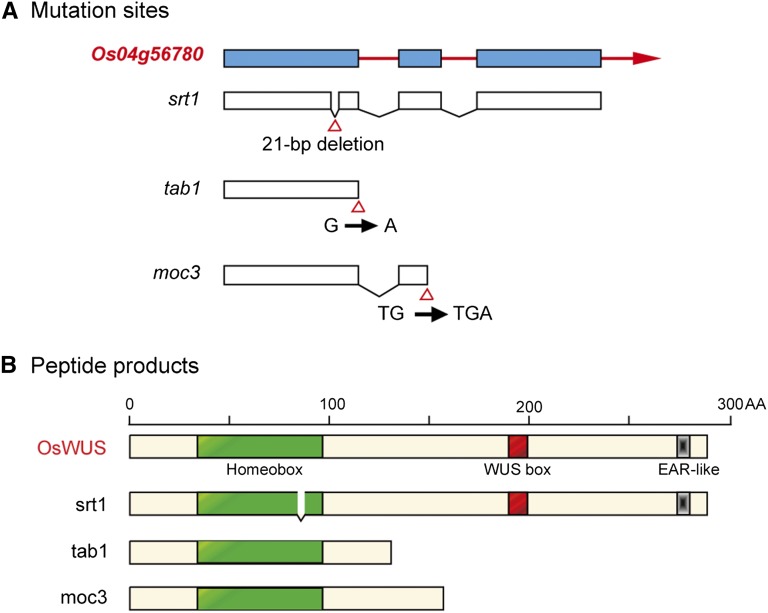
Different mutation sites of OsWUS in the mutant *srt1*, *tab1-1* (Tanaka *et al.* 2015) and *moc3-1* (Lu *et al.* 2015). (A) DNA mutation sites of gene *Os04g56780*. (B) Peptide mutation sites of Os4g56780. srt1 has mutant homeobox and intact WUS box and EAR-like box. However, *tab1-1* and moc3-1 have intact homeobox but without WUS box and EAR-like box.

OsWUS possesses three conserved domains, the homeobox, WUS box, and EAR-like box (Figure 7C), of which the biological functions have not been studied. In both *tab1-1* and moc3-1, the homeobox remains intact, but the whole C-terminal half of the protein containing the WUS box and EAR-like box are not synthesized (Figure 8). Therefore, the functions of *tab1-1* and moc3-1 are likely to be completely lost. In contrast, srt1 retains almost the whole WUS protein except for the short deletion within the homeobox (Figure 8). Protein 3D modeling analysis of the N-terminal region revealed that the homeobox of srt1 lacks a short β-strand, suggesting that a small alteration of the homeobox may destroy the function of the protein. As compared to the other two mutants, the defects in *srt1* were almost as severe as those in *tab1-1* and *moc3-1*, especially for the phenotypes of tillering inhibition and female sterility. The fact that a mutation of the homeobox can result in almost complete loss-of-function indicates that the homeobox is pivotal to the biological function of OsWUS.

In *Arabidopsis*, a loss-of-function mutation in *AtWUS* results in the development of defective flowers lacking most of the central floral organs and having only a single central stamen (Mayer *et al.* 1998). Interestingly, the *Arabidopsis* mutant *wus-3*, which contains a proline to leucine missense mutation in the HD, also produces abnormal flowers, with about three stamens per flower. This suggests that the homeobox is also an important domain for the full function of AtWUS in *Arabidopsis*.

In plants, homeodomain (HD)-containing proteins have been implicated in various developmental processes, such as flower and root development, cell fate specification in meristems, and establishment of leaf polarity (Benfey and Weigel 2001). Analysis of 350 WOX proteins from 50 species of the plant kingdom reveals that all WOX members contain three highly conserved residues in the homeodomain: L (145), I (152), and V (157) (Lian *et al.* 2014). In the 3D structures of AtWUS and OsWUS, these three amino acid residues form an angle at 110.19°, indicating that the three residues may have crucial roles in maintaining the primary function of WUS proteins (Lian *et al.* 2014). The deletion of the seven amino acid residues (KNVFYWF) in srt1 (Figure 8) includes residue V (157). It is speculated that absence of this key residue may result in structural alteration and functional loss of OsWUS. Further characterization of the DNA-binding properties of srt1 and comparison with those of animal homeobox proteins may help decipher the molecular mechanisms underlying the function of WUS proteins in plants.

## References

[bib1] Benfey P. N. Weigel D., 2001 Transcriptional networks controlling plant development. Plant Physiol. 125: 109–111. 11154309 10.1104/pp.125.1.109 PMC1539338

[bib2] Brand U. Fletcher J. C. Hobe M. Meyerowitz E. M. Simon R., 2000 Dependence of stem cell fate in *Arabidopsis* on a feedback loop regulated by *CLV3* activity. Science 289: 617–619. 10915624 10.1126/science.289.5479.617

[bib3] Chen C. C. Hwang J. K. Yang J. M., 2006 (PS)^2^: protein structure prediction server. Nucleic Acids Res. 34: W152–W157. 16844981 10.1093/nar/gkl187 PMC1538880

[bib4] Deyhle F. Sarkar A. K. Tucker E. J. Laux T., 2007 *WUSCHEL* regulates cell differentiation during anther development. Dev. Biol. 302: 154–159. 17027956 10.1016/j.ydbio.2006.09.013

[bib5] Garrison E. Marth G., 2012 Haplotype-based variant detection from short-read sequencing. ArXiv e-prints 1207: 3907.

[bib6] Gehring W. J. Afflter M. Burglin T., 1994 Homeodomain proteins. Annu. Rev. Biochem. 63: 487–526. 7979246 10.1146/annurev.bi.63.070194.002415

[bib7] Groß-Hardt R. Lenhard M. Laux T., 2002 *WUSCHEL* signaling functions in interregional communication during *Arabidopsis* ovule development. Genes Dev. 16: 1129–1138. 12000795 10.1101/gad.225202 PMC186242

[bib8] Ikeda M. Mitsuda N. Ohme-Takagi M., 2009 *Arabidopsis* WUSCHEL is a bifuctional transcription factor that acts as a repressor in stem cell regulation and as an activator in floral patterning. Plant Cell 21: 3493–3505. 19897670 10.1105/tpc.109.069997 PMC2798335

[bib9] Kieffer M. Stern Y. Cook H. Clerici E. Maulbetsch C., 2006 Analysis of the transcription factor WUSCHEL and its functional homologue in *Antirrhinum* reveals a potential mechanism for their roles in meristem maintenance. Plant Cell 18: 560–573. 16461579 10.1105/tpc.105.039107 PMC1383633

[bib10] Lander E. S. Green P. Abrahamson J. Barlow A. Daly M. J., 1987 MAPMAKER: an interactive computer package for constructing primary genetic linkage maps of experimental and natural populations. Genomics 1: 174–181. 3692487 10.1016/0888-7543(87)90010-3

[bib11] Laux T. Mayer K. F. X. Berger J. Jürgens G., 1996 The *WUSCHEL* gene is required for shoot and floral meristem integrity in *Arabidopsis*. Development 122: 87–96. 8565856 10.1242/dev.122.1.87

[bib12] Lenhard M. Bohnert A. Jürgens G. Laux T., 2001 Termination of stem cell maintenance in *Arabidopsis*floral meristems by interactions between *WUSCHEL* and *AGAMOUS*. Cell 105: 805–814. 11440722 10.1016/s0092-8674(01)00390-7

[bib30] Li H. Durbin R., 2009a Fast and accurate short read alignment with Burrows-Wheeler transform. Bioinformatics 25: 1754–1760. 19451168 10.1093/bioinformatics/btp324 PMC2705234

[bib13] Li H. Handsaker B. Wysoker A. Fennell T. Ruan J., 2009b The sequence alignment/map format and SAMtools. Bioinformatics 25: 2078–2079. 19505943 10.1093/bioinformatics/btp352 PMC2723002

[bib14] Lian G. Ding Z. Wang Q. Zhang D. Xu J., 2014 Origins and evolution of WUSCHEL-related homeobox protein family in plant kingdom. The Scientific World Journal Article ID: 534140. 10.1155/2014/534140 PMC3913392 24511289

[bib15] Lohmann J. Hong R. L. Hobe M. Busch M. A. Parcy F., 2001 A molecular link between stem cell regulation and floral patterning in *Arabidopsis*. Cell 106: 793–803. 11440721 10.1016/s0092-8674(01)00384-1

[bib16] Lu Z. Shao G. Xiong J. Jiao Y. Wang J., 2015 *MONOCULM 3*, an ortholog of *WUSCHEL* in rice, is required for tiller bud formation. J. Genet. Genomics 42: 71–78. 25697101 10.1016/j.jgg.2014.12.005

[bib17] Mayer K. F. X. Schoof H. Haecker A. Lenhard M. Jürgens G., 1998 Role of *WUSCHEL* in regulating stem cell fate in the *Arabidopsis* shoot meristem. Cell 95: 805–815. 9865698 10.1016/s0092-8674(00)81703-1

[bib18] Murray M. G. Thompson W. F., 1980 Rapid isolation of high molecular weight plant DNA. Nucleic Acids Res. 8: 4321–4325. 7433111 10.1093/nar/8.19.4321 PMC324241

[bib19] Nardmann J. Werr W., 2006 The shoot stem cell niche in angiosperms: expression patterns of *WUS* orthologues in rice and maize imply major modifications in the course of mono- and dicot evolution. Mol. Biol. Evol. 23: 2492–2504. 16987950 10.1093/molbev/msl125

[bib20] Ohta M. Matsui K. Hiratsu K. Shinshi H. Ohme-Takagi M., 2009 Repression domains of class II ERF transcriptional repressors share an essential motif for active repression. Plant Cell 13: 1959–1968. 11487705 10.1105/TPC.010127 PMC139139

[bib21] Pabo C. O. Sauer R. T., 1984 Protein-DNA recognition. Annu. Rev. Biochem. 53: 293–321. 6236744 10.1146/annurev.bi.53.070184.001453

[bib22] Schoof H. Lenhard M. Haecker A. Mayer K. F. X. Jürgens D., 2000 The stem cell population of *Arabidopsis* shoot meristems is maintained by a regulatory loop between the *CLAVATA* and *WUSCHEL* genes. Cell 100: 635–644. 10761929 10.1016/s0092-8674(00)80700-x

[bib23] Sieber P. Gheyselinck J. Groß-Hardt R. Laux T. Grooniklaus U., 2004 Pattern formation during early ovule development in *Arabidopsis thaliana*. Dev. Biol. 273: 321–334. 15328016 10.1016/j.ydbio.2004.05.037

[bib24] Tanaka W. Ohmori Y. Ushijima T. Matsusaka H. Matsushita T., 2015 Axillary meristem formation in rice requires the *WUSCHEL* ortholog *TILLERS ABSENT1*. Plant Cell 27: 1173–1184. 25841039 10.1105/tpc.15.00074 PMC4558701

[bib25] van der Graaff E. Laux T. Rensing S. A., 2009 The WUS homeobox-containing (WOX) protein family. Genome Biol. 10: 248. 20067590 10.1186/gb-2009-10-12-248 PMC2812940

[bib26] Xu S. Tao Y. Yang Z. Chu J., 2002 A simple and rapid method used for silver staining and gel preservation. (in Chinese) Hereditas 24: 335–336. 16126695

[bib27] Yadav R. K. Perales M. Gruel J. Grike T. Jonssön H., 2011 WUSCHEL protein movement mediates stem cell homeostasis in the *Arabidopsis* shoot apex. Genes Dev. 25: 2025–2030. 21979915 10.1101/gad.17258511 PMC3197201

[bib28] Zhang X. Zong J. Liu J. H. Yin J. Y. Zhang D. B., 2010 Genome-wide analysis of WOX gene family in rice, sorghum, maize, *Arabidopsis* and poplar. J. Integr. Plant Biol. 52: 1016–1026. 20977659 10.1111/j.1744-7909.2010.00982.x

